# Uveal melanoma presenting as cataract and staphyloma

**DOI:** 10.4103/0301-4738.49398

**Published:** 2009

**Authors:** Vikas Khetan, Kshanada Gupta, E. Ravindra Mohan, Lingam Gopal

**Affiliations:** 1 Department of Vitreoretina and Ocular Oncology, Medical Research Foundation, Sankara Nethralaya, Chennai, India; 2 Department of Oculoplasty, Medical Research Foundation, Sankara Nethralaya, Chennai, India

**Keywords:** Complicated cataract, extended enucleation, staphyloma, uveal melanoma

## Abstract

Blind eyes can harbor a choroidal melanoma. We report a case of uveal melanoma presenting as staphyloma and complicated cataract in a 45-year-old female. The left eye was blind for six months. She underwent comprehensive ocular examination but fundus examination was precluded due to total cataract. The ultrasound of the eye showed a large mass filling the superior, nasal and inferonasal vitreous cavity with high surface reflectivity and low to moderate internal reflectivity. Magnetic resonance imaging (MRI) confirmed the diagnosis of choroidal melanoma. The patient underwent extended enucleation and histopathology was consistent with uveal melanoma.

Uveal melanoma is the most common primary malignant intraocular neoplasm of Caucasian adults.[1] Uveal melanomas have an overall mortality of about 30–50% within 10 years from initial diagnosis and treatment. Deaths are mostly secondary to distant metastases. Hence, the importance of having a high suspicion of this entity, especially in blind eyes with no fundus view. We report a case of a uveal melanoma presenting as cataract and staphyloma in a blind eye

## Case Report

**Figures 1a and b:**
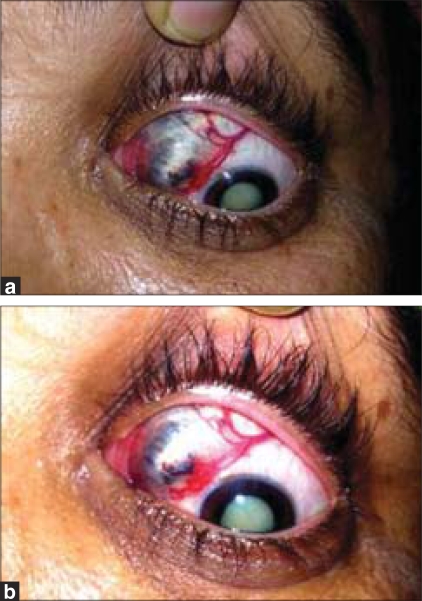
Clinical appearance of the right eye

A 45-year-old lady presented to us with a history of painless swelling in the left eye associated with decrease in vision since six months. On ophthalmic examination, she had a best corrected visual acuity (BCVA) of 20/20 in the right eye and light perception with inaccurate projection of rays in the left eye. The anterior segment examination in the right eye was within normal limits. In the left eye there was a staphyloma in the superonasal quadrant, close to the limbus measuring 6 × 6 mm. It was irregular in shape, brown in color with overlying scleral thinning and a sentinel vessel temporal to the staphyloma [Figs. 1a, b]. Iris showed 360° rubeosis and the lens was totally cataractous. The pupil was fixed and dilated. The extraocular motility was restricted on elevation. Applanation tonometry was 36mm of Hg. Gonioscopy revealed 360 degrees closed angles. There was no fundal glow in the left eye. Fundus examination of right eye showed a disc with 0.6 cup-disc ratio, temporal pallor and inferior neuroretinal rim thinning.

The ultrasound examination of the left eye showed a large mass filling the superior, nasal and inferonasal vitreous cavity with high surface reflectivity and low to moderate internal reflectivity. The mass was extending posteriorly and involving the optic nerve head. Secondary retinal detachment was noted over the mass [Fig. 3]. It was however, inconclusive for melanoma.

**Figures 2 a and b:**
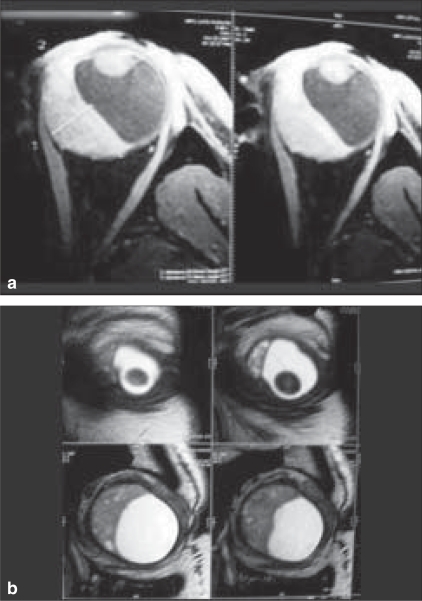
The magnetic resonance imaging findings

Magnetic resonance imaging (MRI) of the brain and orbit revealed an intraocular enhancing choroidal mass lesion extending from just posterior to the ciliary body along the nasal quadrant up to the optic disc and extending to the temporal side of the optic disc. It displayed hyperintense signal in T1W1 and hypointense in T2W1 with respect to vitreous with fairly homogenous contrast enhancement [Figs. 2a, b]. A diagnosis of choroidal melanoma was made. X-ray chest was normal. Ultrasound of the abdomen showed a nodule in the liver which was inconclusive. Liver function tests were normal.

**Figure 3:**
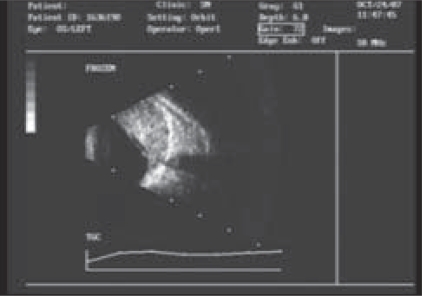
The ultrasonographic findings

The patient underwent a modified extended enucleation under general anesthesia.

**Figure 4:**
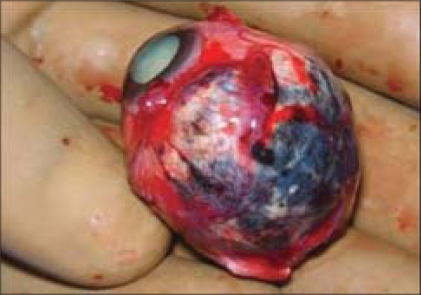
The gross postoperative appearance of the enucleated eyeball

During enucleation care was taken not to rupture the globe. Meticulous dissection was carried out in the staphylomatous area. The enucleated eyeball showed almost a total loss of scleral fibers in the entire nasal quadrant [Fig. 4].

**Figure 5:**
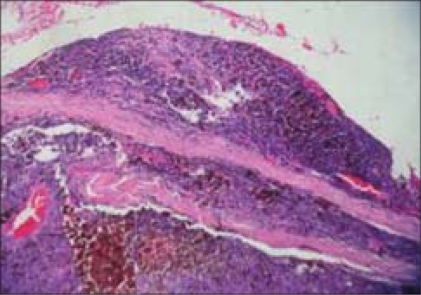
Histopathological findings of the enucleated eyeball (H&E, ×20)

**Figure 6:**
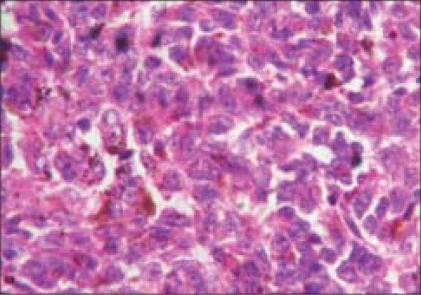
Histopathological findings under high magnification (H&E, ×40)

The histopathological examination showed a pigmented tumor mass arising from the anterior part of the choroid which was composed of pigment-bearing spindle cells and epithelioid cells. Multiple mitotic figures were seen [Figs. 5, 6]. Extrascleral extension was also noted histopathologically. Histopathological diagnosis of malignant melanoma of choroid of the mixed cell type was made. Six weeks postoperatively, the orbit was free of any tumor recurrence but the liver nodule enlarged and was confirmed as metastasis. The patient was advised palliative chemotherapy.

## Discussion

Uveal melanoma is the most common primary malignant intraocular neoplasm of Caucasian adults. Annual incidence in Caucasians is seven to eight new cases per million persons.[1] Choroidal melanoma is rare in African blacks and intermediate in frequency in other racial groups. Average age is 55–60 years in most large series. Incidence is slightly higher in men than women.[2]

A case of uveal melanoma reporting as panophthalmitis has been described earlier, however, this type of presentation is very rare.[3] In a previous review of the literature of melanoma in blind eyes Patricia *et al*., have presented a total of 11 cases including two of their own.[4] They have also stressed the need for ultrasound examination in such eyes.[4–10] However, most of those eyes were painful as well. Despite neovascular glaucoma, our case had no pain and hence the patient did not seek medical attention.

This case highlights the need to be alert to such a possibility in cases presenting essentially as a blind eye with staphyloma and complicated cataract. Surgical removal of such a staphylomatous eye should be done with care to avoid accidental globe rupture. Liberal canthotomy and meticulous conjunctival dissection near the staphylomatous area helped avoid such a catastrophe.

## Conclusion

Uveal melanoma should also be suspected in patients with staphyloma and cataract precluding fundus evaluation. An ultrasound should be performed in all cases presenting to us as blind eyes without a known cause.

## References

[1] Egan KM, Seddon JM, Glynn RJ, Gragoudas ES, Albert DM (1988). Epidemiologic aspects of uveal melanoma. Surv Ophthalmol.

[2] Iscovich J, Ackerman C, Andreev H, Pe'er J, Steinitz R (1995). An epidemiological study of posterior uveal melanoma in Israel, 1961-1989. Int J Cancer.

[3] Sood NN, Ratnaraj A (1968). Malignant melanoma of Choroid presenting as panophthalmitis. J All India Ophthalmol Soc.

[4] Pereira PR, Odashiro AN, Souza Filho JP, Saraiva VS, Camoriano DG, Burnier MN Jr (2006). Malignancy in the blind painful eye-report of two cases and literature review. Diagn Pathol.

[5] De Gottrau P, Holbach LM, Naumann GO (1993). Acute glaucoma: First manifestation of malignant melanoma of the choroid. J Fr Ophtalmol.

[6] Bianciotto C, Saornil MA, Muiños Y, Méndez MC, Blanco G, Frutos-Baraja JM (2005). Ocular hypertension as the principal indicator of onset of uveal melanoma. Arch Soc Esp Oftalmol.

[7] Shields JA, Augsburger JJ (1985). Cataract surgery and intraocular lenses in patients with unsuspected malignant melanoma of the ciliary body and choroid. Ophthalmology.

[8] O'Leary SW, Ramsey MS (1990). Unsuspected uveal melanoma diagnosed after cataract extraction. Can J Ophthalmol.

[9] Chess J, Henkind P, Albert DM, Gragoudas ES, Reidel K, Weiss J (1985). Uveal melanoma presenting after cataract extraction with intraocular lens Implantation. Ophthalmology.

[10] Escalona-Benz E, Benz MS, Briggs JW, Budenz DL, Parrish RK, Murray TG (2003). Uveal melanoma presenting as acute angleclosure glaucoma: Report of two cases. Am J Ophthalmol.

